# Multiple Approaches to the Investigation of Cell Assembly in Memory Research—Present and Future

**DOI:** 10.3389/fnsys.2018.00021

**Published:** 2018-05-25

**Authors:** Yoshio Sakurai, Yuma Osako, Yuta Tanisumi, Eriko Ishihara, Junya Hirokawa, Hiroyuki Manabe

**Affiliations:** Laboratory of Neural Information, Graduate School of Brain Science, Doshisha University, Kyoto, Japan

**Keywords:** cell assembly, multineuronal recording, optogenetics, live cell imaging, brain-machine interface

## Abstract

In this review article we focus on research methodologies for detecting the actual activity of cell assemblies, which are populations of functionally connected neurons that encode information in the brain. We introduce and discuss traditional and novel experimental methods and those currently in development and briefly discuss their advantages and disadvantages for the detection of cell-assembly activity. First, we introduce the electrophysiological method, i.e., multineuronal recording, and review former and recent examples of studies showing models of dynamic coding by cell assemblies in behaving rodents and monkeys. We also discuss how the firing correlation of two neurons reflects the firing synchrony among the numerous surrounding neurons that constitute cell assemblies. Second, we review the recent outstanding studies that used the novel method of optogenetics to show causal relationships between cell-assembly activity and behavioral change. Third, we review the most recently developed method of live-cell imaging, which facilitates the simultaneous observation of firings of a large number of neurons in behaving rodents. Currently, all these available methods have both advantages and disadvantages, and no single measurement method can directly and precisely detect the actual activity of cell assemblies. The best strategy is to combine the available methods and utilize each of their advantages with the technique of operant conditioning of multiple-task behaviors in animals and, if necessary, with brain–machine interface technology to verify the accuracy of neural information detected as cell-assembly activity.

## Introduction

The hypothesis of cell assemblies, functional groups of neurons, was first proposed by the psychologist Hebb (1949). This hypothesis arising from psychological experiments and insights during the days, when neuronal activities could not be recorded, is now much more noticed and driving the field of the neuroscience of memory. The starting point for Hebb’s awareness of issues was the “organization of perception” and not memory. Organization was originally one of the functions proposed by Gestalt psychology. For example, triangles of various shapes and sizes have different physical characteristics; thus, different stimulation points on the retina, as well as different neurons in the primary visual cortex, can be ignited. However, it is a function that is perceived as a unit called the “triangle.” Hebb tried to explain the organization of this perception with the findings of Lorente de Nò (1934), who suggested that feedback circuits exist in the nervous system. The center consists of a functional neuron population that is created occasionally because of enhanced functional synaptic coupling between simultaneously active neurons (Hebb synapse). This functional neuron population was termed cell assembly. In other words, even if different neurons fire, the same perception (e.g., triangle) will occur if it leads to the activity of the same cell assembly. If cell assemblies encode information, i.e., what is represented and/or conveyed in the brain to generate activity in mind, in this way, it is possible that the perception and appearance of an entire image are established by using fragmented stimuli only, and concepts are formed from various pieces of information.

Similar principles can be applied to explain the formation and transformation of memories, because memories are closely connected with perception and images (Hebb, 1949). As many cognitive psychology experiments have already shown, memory is not a simple process of consolidating experienced events but dynamic processes of organizing, modifying and relating the experienced events with stored and new information (e.g., Schank, 1999). For example, none of behavioral data has proved that all memory storage permanently remain and unchanged and “false memory” can be created sometime easily (Loftus and Loftus, 1980; Loftus, 1997). Therefore, memory and information processing are inseparable and “Memory is determined by information processing” (Squire, 1987, p. 124). Such dynamic coding and processing of information in memory, not just coding and retaining the information as an engram, has been assumed to be explained by the function of cell assembly. Actually, Hebb described “There may, then, be a memory trace that is wholly a function of a pattern of neural activity, independent of any structural change.” (Hebb, 1949, p. 61). If the cell assembly only forms a rigid engram, it is the same as saving the information on a hard disk, and information processing unique to memory, such as the formation of memory by association, the recollection of memories, and the dynamic organization of memory, cannot be explained.

## Experimental Detection 1—Multineuronal Recording

At present, the concept that is widely accepted for cell assembly can be summarized as follows. It is a functional neuron population that is formed at any time according to information, and neurons constituting the group exhibit synchronous firing. Individual neurons overlap and participate in different cell assemblies, i.e., each neuron exhibits functional overlap. Moreover, neurons change synchronous firing within a group or among groups according to necessary information, i.e., there is connection dynamics among neurons, and occasionally form large and small cell assemblies. The synaptic strength connecting neurons also occasionally changes according to Hebb’s rule.

**Figure 1:**
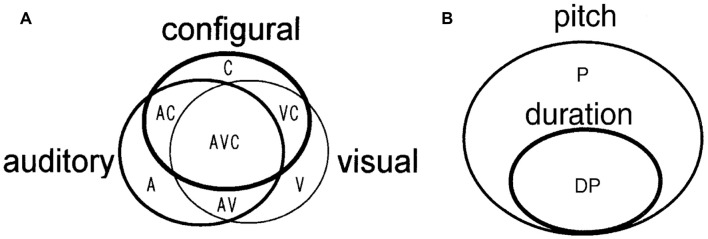
**(A)** A model of cell assemblies presented in Sakurai (1996); which shows the functional overlap of individual neurons and functional connectivity of the neurons. The circles represent cell assemblies, each of which codes the auditory, visual, or configural discrimination task. These cell assemblies consist of task-related single neurons (A, V, C, AV, AC, VC, and AVC). A, Neurons related to the auditory discrimination task (A task); V, neurons related to the visual discrimination task (V task); C, neurons related to the configural auditory–visual discrimination task (C task); AV, neurons related to A and V tasks; AC, neurons related to A and C tasks; VC, neurons related to V and C tasks; AVC, neurons related to all tasks. **(B)** A model of cell assemblies presented in Sakurai (2002). The circles represent cell assemblies, each of which codes the pitch or duration discrimination task. These cell assemblies consist of the task-related single neurons (P and DP). P, neurons related to the pitch discrimination task. DP, neurons related to both duration and pitch discrimination tasks. From Sakurai (1996; 2002) with permission.

Thus, the most important characteristic of a cell assembly is “to be formed at any time according to necessary information.” To experimentally detect this process, multineuronal activity should be simultaneously recorded from a brain that is coding and processing multiple and changing information, such as animals performing complicated tasks or multiple tasks (Sakurai, 1996, 1999; Roudi et al., 2015). In our previous research, we recorded multineuronal activity from the hippocampus and the neocortex of the same rat performing different tasks. We detected two features that suggested the existence of cell assemblies, i.e., the functional overlap of individual neurons seen between different tasks and the dynamics of functional coupling between neurons occurring between tasks. Therefore, we reported that each cell assembly codes for the type of task being performed (Sakurai, 1996, 2002; Figure 1).

Cell assembly is a functional group that is created as needed between a small number of neurons in close distance or between large numbers of separate neurons (Eichenbaum, 1993). We noticed that it was difficult to detect the cell assembly made by adjacent neurons. This is because it was almost impossible to accurately separate the firing of neighboring multiple neurons and, therefore, to detect synchronous firing among the neurons by the extracellular recording method, which records neuronal activity for a long time in free-moving animals. However, it became possible by using the spike-sorting method utilizing independent component analysis (ICA; Takahashi et al., 2003). It was demonstrated that approximately 80% of the neuron pairs in the prefrontal cortex in monkeys showed firing correlation and jitters of spike times of 1–5 ms. Furthermore, the firing correlation of some of the neuron pairs appeared or disappeared according to the type of the memory task being performed (Sakurai and Takahashi, 2006). The reason for the substantial proportion of neuron pairs showing firing correlation (~80%) might be due to the detection of firings from closely neighboring neurons by spike sorting with ICA. Recent live-cell imaging studies (e.g., Dombeck et al., 2010) reported that many of the neighboring neurons were firing together. This result shows that many neighboring neurons of the frontal association area are functioning by making local cell assemblies (Sakurai and Takahashi, 2008).

**Figure 2:**
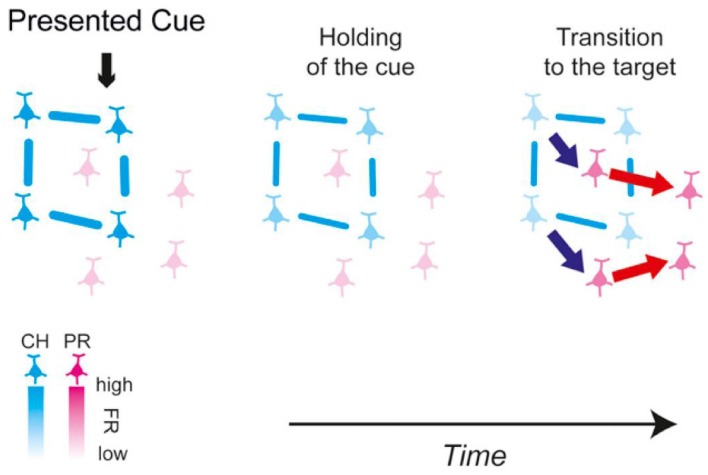
Schematic of the functional microcircuitry in the perirhinal cortex in retrieval of object association memory presented in Hirabayashi et al. (2013). Cyan and magenta neurons are cue-holding and pair-recall neurons, respectively. The blue and red arrows between neurons depict the directed interactions identified in the study. The lines between neurons represent functional couplings. The results suggest that during memory retrieval, cue information is transmitted from the CH cell assembly to the PR cell assembly to convert the representation in the microcircuit from the cue to the sought target. From Hirabayashi et al. (2013) with permission.

The above experiments identified that cell assemblies are generated at any time depending on the information processing of memory; however, the details of information conversion between cell assemblies during the processing of memory were completely unknown. However, Miyashita’s Lab in University of Tokyo successfully showed the process of information conversion between cell assemblies as being responsible for retaining and recalling information. The team used a combination of a monkey’s paired association memory task with fractal figures, the recording of multineuron activity, and Granger Causality Analysis to demonstrate that recalling the other figure of a pair could represent information conversion between cell assemblies (Takeuchi et al., 2011; Hirabayashi et al., 2013). In these studies, they first identified the flow of information between the cortical layers in the temporal lobe electrophysiologically. Then, they demonstrated that the signal flows through the cortical layer IV → layer II and layer III→layer V/VI when the monkey viewed the figures as cues that should be memorized and that the signal flows conversely through layer V/VI → layer II/III when recalling the figures (Takeuchi et al., 2011). They simultaneously recorded the activity of multiple neurons in different cortical layers and identified the signal flows between the recorded neurons. They also demonstrated that the activity of the neuron population (cell assembly), which retains the image of the cue figures, activates the cell assembly that codes the figure to be recalled and these cell assemblies are partially overlapped (Hirabayashi et al., 2013; Figure 2). These studies experimentally show the information conversion between cell assemblies during the information processing of memory, which is a very big progress.

Besides the above studies of neocortical cell assemblies, the hippocampal literature of cell-assembly research has made great progress. In addition to the well-known literature of hippocampal place cells and cell assembly (e.g., Dragoi and Buzsáki, 2006), various new frameworks of electrophysiological analyses and simulation showed the existence of cell assemblies in the rat hippocampus (e.g., Lopes-dos-Santos et al., 2013). Recent studies in rats suggest that the hippocampal cell assembly encodes different information one after another according to the theta rhythm when processing specific memory (Terada et al., 2017).

## What Two-Neuron Correlation Reflects

On the basis of the above experimental examples of multineuronal recording, it was concluded that the correlation between two neurons constitutes evidence of the activity of cell assembly. Although several methods have been devised and used in experiments to detect and display correlations among three or more neurons at once (Holscher and Munk, 2009; Gruen and Rotter, 2010), among which Hidden Markov modeling (e.g., Eddy, 2004) might become a standard, many researches have adopted a convenient method of sequentially selecting two neurons from multiple neurons and examining the respective synchronous firings (e.g., Tatsuno, 2015).

**Figure 3:**
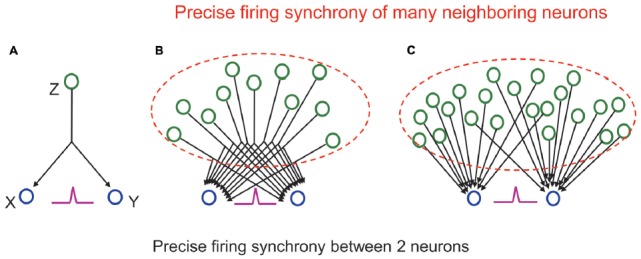
**(A)** A cross-correlogram showing a sharp central peak (purple) indicating precise firing synchrony between the two neurons reflects not firing of a driving neuron, which sends a common input to them, but precise firing synchrony of many neighboring neurons (in dashed lines) sending many common inputs **(B)** or many individual inputs **(C)** to them. See main text for detail.

However, it is often suggested that the neuron population containing the two selected neurons does not necessarily have a functional connection with each other even if the two neurons are synchronously firing. Functional connection (connectivity) is different from structural synaptic connection and defined as the temporal correlation of activity between distributed neurons and neuronal groups, expressed as deviation from statistical independence (Friston et al., 1993; Fingelkurts et al., 2005). Then, it should be asked what does it mean for two neurons to be “functionally connected”? (Stevenson et al., 2008). Aertsen et al. (1989) suggests that it is impossible to uniquely determine the “true” functional connectivity of a network without recording from all elements, because unobserved elements in the network can always confound connectivity estimates (Horwitz, 2003). Typical such elements are “unobserved common inputs” (Stevenson et al., 2008) to the neurons showing firing synchrony to each other. If you assume that there is a neuron Z sending output to both neurons X and Y (common input), these two neurons will fire synchronously when Z fires and activates them (Figure 3A; see Ratte et al., 2013 for more discussion on physiology of common inputs). In the end, the synchronous firing of X and Y might only reflect the firing of one neuron Z and does not mean any “true” functional connection to each other.

However, we should remember that the functional coupling between individual neurons is originally very weak (Schneidman et al., 2006). The probability that one neuron, which receives input from another neuron, fires when the output neuron fires is very low. Such probability is called synapse “contribution,” and it is known to be only a few percent in most synapses in cortical areas (Abeles, 1988). As shown in Figure 3A, even if Z fires 100 times, the number of times X or Y fires is only a few times, and the number of times X and Y fire together is much smaller. In other words, it is actually impossible that the exact synchronous firing of neurons X and Y is controlled only by the firing of neuron Z. In reality, one neuron has thousands of synaptic inputs. Therefore, even if the input from one neuron does not have a large effect, the next neuron will fire well, and the neuronal signal will be reliably transmitted when many of the inputs act simultaneously. When synchronous firing is seen between two neurons, it reflects that a number of neurons have functional connections to the two neurons that are outputting simultaneously (Figures 3B,C). Consequently, synchronous firing between two neurons means that a huge number of surrounding neurons are also synchronously firing, and these neurons have functional connections to the neurons that exist behind them.

Multineuronal recording is the only method that can measure spikes and their synchrony in neuron activity in real time. Therefore, the recording and analytical methods of multineuronal activities are still developing (Lopes-dos-Santos et al., 2013; Roudi et al., 2015). On the other hand, the number of neurons that can be recorded simultaneously is limited, the exact position of each neuron is unknown, and the distance between neurons can be estimated only as far or close, though the exact physical location of recorded neurons may not be important in understanding the function of a neuronal group. However, multi-contact silicon probes and similar devices have been used by a number of laboratories to examine anatomical organization and/or topographical arrangement of neurons and their functional responses to some extent. For example, Hampson et al. (1999) reported the anatomical organization of dorsal hippocampal neurons according to spatial/non-spatial events and Sakata and Harris (2009) suggested the laminar structure of populations of neurons in the auditory cortex in relation to spontaneous and sensory-evoked activity.

## Experimental Detection 2—Optogenetics

For correspondence between memory and cell assembly, significant progress was made recently by experiments that reactivated the cell assemblies that encode memory by utilizing optogenetics. As one of the pioneering works in Tonegawa’s Lab in RIKEN, Liu et al. (2012) conducted a contextual fear conditioning experiment whereby an electric shock within a specific experiment box was applied to c-fos-tTA genetically modified mice. The virus vector TRE-channelrhodopsin-2 (ChR2)-EYFP was injected into the hippocampus of these mice, and ChR2 was synthesized only in the neurons that were active in contextual fear conditioning. Thereafter, the day after conditioning, when the mice were placed in the same experiment box as the previous day, they showed a freezing response. Conversely, when the mice were placed in a different experiment box, they showed no freezing response. In other words, the mice memorized the experiment box and the surrounding environment that they had experienced fear in. However, when light stimulation was applied to the hippocampus of mice placed in an experiment box that was different from the previous day, the neuron population that was active during the conditioning procedure on the previous day was activated again, and the mice showed a fear response. This result indicates that the neuron population, which was active during fear conditioning, is a cell assembly that codes for fear memory.

**Figure 4:**
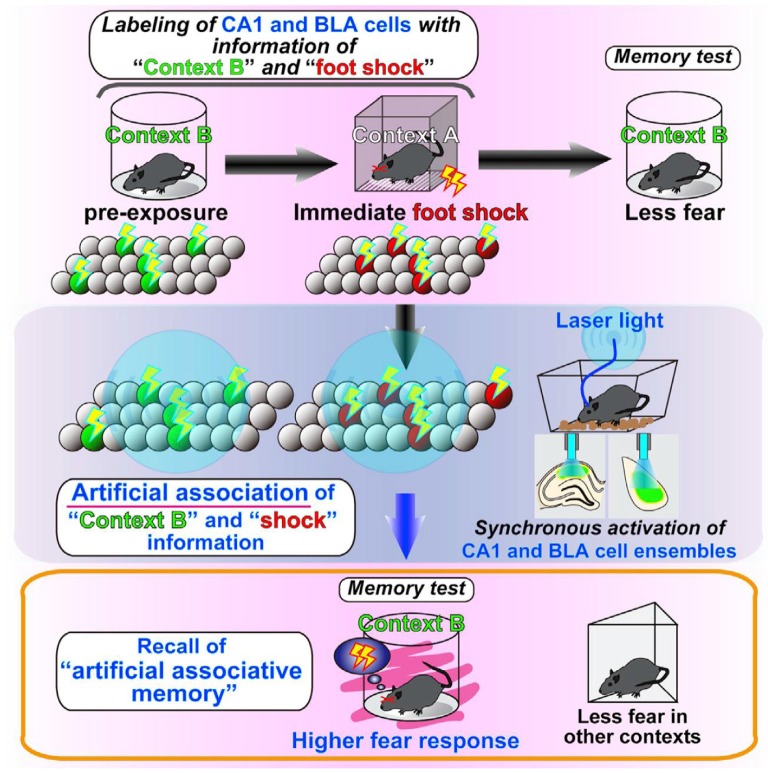
Illustration of the results presented in Ohkawa et al. (2015). They found that the coincident firing of distinct cell assemblies generates a link between these cell assemblies, thus leading to an association of originally independent memory episodes. From Ohkawa et al. (2015) with permission.

**Figure 5:**
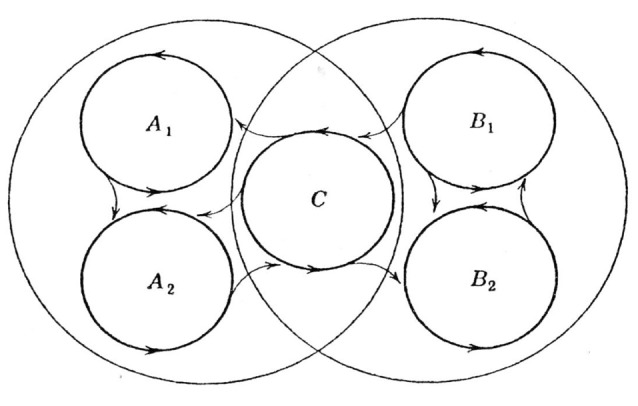
Schematic of Hebb’s postulate presented in Hebb (1949). This schematic illustrates the possibility that a subsystem C may act as a link between two systems (conceptual complexes). One concept is represented by A1, A2 and C, and the second concept is represented by B1, B2 and C. The two systems have a subsystem C in common to provide a basis of prompt association to generate a new system (a new concept). From Hebb (1949) with permission.

The reactivation experiments of cell assemblies that code for such stored information have further advanced the field. By using the same c-fos-tTA genetically modified mice as before, Inokuchi’s Lab in Toyama University conducted a unique experiment (Ohkawa et al., 2015). They synthesized ChR2 in the neuron population X of the hippocampus and the amygdala, which was active when the mouse was placed in a safe cylindrical box, and in the neuron population Y of the hippocampus and the amygdala, which was active when the mouse was placed briefly in a cubic box in which it experienced an electric shock. The following day, when the mouse was in the home cage, light stimulation was given to the hippocampus and the amygdala to activate neuronal populations X and Y simultaneously. Thereafter, even though the mouse was placed in the cylindrical box, in which the mouse never received an electric shock, it showed a fear response (Figure 4). If population X overrides population Y by their simultaneous activation, the mouse will also show a fear response in the cylindrical box. However, populations X and Y are independent cell assemblies that code different memories, and there is no up-and-down relationship between them. Therefore, it is reasonable to conclude that by simultaneously activating different cell assemblies that respectively code the two unrelated memories of the cylindrical box and fear experience, a new cell assembly that associates those memories was successfully created. This finding is quite interesting because it is consistent with Hebb’s theory (Figure 5), which states that new information is created by associating different cell assemblies by synchronous activities and becoming a new large cell assembly, thus leading to “concept” formation.

The reactivation experiments of such cell assemblies are possible not only by optogenetics but also by other genetic modifications and pharmacological manipulations (Matsuo, 2015; Yoshii et al., 2016). Therefore, there is no doubt that further studies of the causal relationship between cell-assembly activity and behavior will further advance. However, a few more considerations are needed to interpret and evaluate the results of optogenetic stimulation studies. First, we should carefully examine the correspondence between behaviorally-triggered patterns of neuronal ensemble activity with that induced by optogenetic stimulation. In addition to such examination of the technical accuracy of the genetic modification and ChR2 synthesis, the neuronal networks in the working brain are continuously changing by experiences and time passage, inevitably causing functional changes of the individual neurons and their networks. Therefore, the activated neurons when the animals were trained and those when optogenetically stimulated later might not have the same functions in a strict sense. Second, a recent advanced study using multineuronal recording with 512-channel tetrode system combined with *Cre*-lox neurogenetics and optogenetics reported that localized optogenetic manipulation disrupted network oscillations and caused changes in single-unit firing patterns in a brain-wide manner (Xie et al., 2016b). This result raises the caution of the interpretation of optogenetically manipulated behaviors, indicating the possibility that optogenetically elicited behaviors are caused not by reactivation of the previous or associated cell assemblies but by newly excited activity patterns in a wide range of the brain.

Third, most studies have so far used behavioral tasks that can be learned in one experience, such as contextual fear conditioning, to instantaneously synthesize ChR2 only for a specific neuron population in the hippocampus. Given that the contextual fear conditioning does depend on the hippocampus, cued fear conditioning depends not on the hippocampus but mainly on the amygdala (e.g., LeDoux, 2000; Wolff et al., 2014) and/or the prelimbic cortex (e.g., Paré and Quirk, 2017). Considering those earlier and elaborated works of fear memory, it could be suggested that the neuronal circuits underlying fear memory for context might be partly related to the amygdala and/or the prelimbic cortex besides the hippocampus. Concerning Ohkawa et al. (2015) introduced above, the simultaneous and repeated stimulation of the respective cell assemblies could consolidate the associative memory—likely represented by a new cell assembly, and the conjunctive representation of context B plus foot shock might exist in a subset of amygdala or prelimbic cortical neurons, whose synaptic connections strengthen as a result of the coincident activation. Further, it could be the consolidation of these synaptic strength changes that promotes more robust fear responses during the subsequent test session.

On the other hand, higher and positive memory is mostly formed by learning with rewards for longer periods and there are many hippocampus-dependent higher learning tasks. Therefore, there is a high possibility that higher memory requires cell-assembly mechanisms different from those for fear memory. In the near future, it will be necessary to find a way to explore the causal relationships between the formation of diverse and higher-order memories by using rewards and cell assembly activities.

In recent years, a new genetically encoded neuron perturbation method, called “chemogenetics,” has been developed to control neuronal firing using small molecules that activate engineered receptors that can be targeted to cell types (Wess et al., 2013; Sternson and Roth, 2014). This method avoids the invasive nature of optogenetics, for which fibers for light stimulation are chronically implanted in the brains, like for electrophysiological recording, to activate or inhibit neurons electrical activity. Receptors with several cellular functions have been developed to facilitate the selective pharmacological control over a diverse range of neuronal signaling and firing for molecularly defined cell types. Chemogenetics has already been used widely to investigate the causal relationship between brain activity and behavior. For example, Vetere et al. (2017) reported that *in vivo* chemogenetic silencing of different network nodes (vertices) impaired fear memory consolidation in mice. This method can reveal specific influences for molecularly defined cell types that are often intermingled with neuronal populations having different functions (Sternson and Roth, 2014), suggesting that it could be applied to detect and activate specific cell assemblies in behaving animals.

## Experimental Detection 3—Live-Cell Imaging

The optogenetics can identify cell assemblies that encode memory by clarifying the causal relationship between the activity of a specific neuron population and behavior change (Tonegawa et al., 2015a,b). However, it is impossible to measure in real time the changes that occur in the cell assembly due to changes in memory. Even the multineuronal recording, already described above, the “real image” or “real structure” of cell assemblies cannot be detected. In other words, we can detect the cell assembly as a static memory engram or activity of a partial group of neurons of it, but we cannot see the dynamics of the full picture of the cell assembly, which changes rapidly according to the information processing of memory.

**Figure 6:**
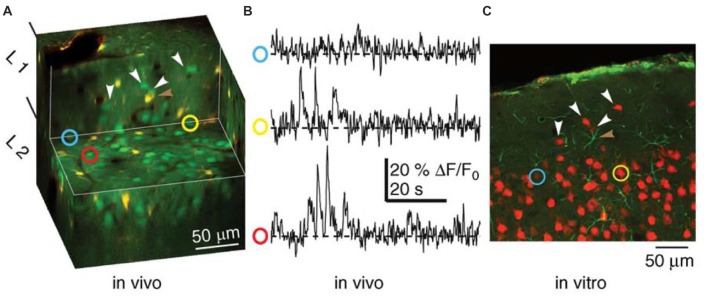
Combined functional and anatomical methods of live-cell imaging for studying cell assemblies (Wallace and Kerr, 2010). Recordings of neuronal activity (Ca^2+^-transients) recorded from a population of neurons using *in vivo* multiphoton microscopy and subsequent identification of the same neurons in a histological section. **(A)** Cut-away, three-dimensional representation of tissue imaged *in vivo*. Neurons appear green and astrocytes appear yellow. The horizontal exposed surface shows the imaging plane from which functional data were collected. **(B)** Ca^2+^ imaging traces from the neurons identified by the yellow, red and blue circles in **(A)**. **(C)** A histological section from the tissue shown in **(A)**, in which two of the neurons from which functional activity was recorded were identified. Neurons appear red, while astrocytes appear green. White arrowheads in **(A,C)** indicate matched neurons. The brown arrowhead highlights an astrocyte visible in both panels. Modified from Wallace and Kerr (2010) with permission.

To compensate for the weakness of the methods of optogenetics and electorophysiology, a measurement method called live-cell imaging was developed recently (Figure 6). This method visualizes individual neuron activity as a function of changes in Ca^2+^ levels by using two-photon microscopy (Wallace and Kerr, 2010; Grienberger and Konnerth, 2012). Several years ago, when it became possible to measure the activity of hundreds of neurons in real time from awake and head-fixed mice “navigating” in virtual environments (Dombeck et al., 2010), this method began to be used for *in vivo* experiments in many labs (e.g., Sato et al., 2016). Although Dombeck et al. (2010) is surely the pioneering study of *in vivo* live-cell imaging and the simultaneous visualization of many place cells in the hippocampus is compelling, comparative evaluation with the electrophysiological place-cell studies should be carefully discussed. A concern of this study is that the virtual navigation limits the degree to which the vestibular system is engaged during navigation, as would be the case during active exploration of mice in a real-world environment. Therefore, the picture of neuronal ensembles supporting virtual navigation may not fully reflect the dynamic nature of cell-assembly responses during real-world navigation.

The method of live-cell imaging itself has a problem. The sampling rate of imaging, i.e., the time resolution, is relatively low. When hundreds of neurons are measured simultaneously, the sampling rate is 30–60 Hz, and single-neuron spikes (–1 ms) cannot be accurately detected. Therefore, synchronization at the submillisecond level between adjacent neurons seen in the prefrontal cortex and hippocampus (Sakurai and Takahashi, 2006; Takahashi and Sakurai, 2009; Diba et al., 2014), i.e., the activity of a local cell assembly (Sakurai and Takahashi, 2008), cannot be detected either. It will be difficult in principle to detect spikes in real time in a manner similar to multineuronal recording because live-cell imaging depends on changes in Ca^2+^ levels. Furthermore, when monitoring the cellular activity of structures below the cortical surface, e.g., the hippocampus, it is necessary to largely remove the overlying cortex by aspiration to enable direct imaging by two-photon microscopy (Denk and Svoboda, 1997). The effects of such large cortical lesions (windows for chronic imaging) on the cellular activity of the subcortical structures should be checked carefully.

Despite the limitations of this technique, as described above, the ability to monitor the activity of a large population of neurons in awake, behaving animals is surely important for further research on cell assemblies. For instance, the pioneering study (Dombeck et al., 2010) collected time-series movies (–64 ms per frame) of fields of view (–200 × 100 μm) in the CA1 region of the hippocampus containing −80 to 100 neurons and optically identified and characterized populations of place cells. The authors determined a correlation between the location of their place fields in the virtual environment and their anatomical location in the local circuit. Such a correlation in a population of neurons is intriguing and cannot be determined by any other techniques using behaving animals.

Recent studies (Driscoll et al., 2017; Grewe et al., 2017) have demonstrated the merits of this technique in detecting both the dynamics and stability of neuronal representations across days in some brain regions. Driscoll et al. (2017) recorded two-photon images and tracked the activity of several hundred cells in the posterior parietal cortex for a month as mice performed a virtual-navigation task. They found that the relationship between cell activity and task features was mostly stable on single days but underwent major reorganization over weeks. Although individual neurons informative about task features (trial type and maze locations) changed across days, the cells’ population activity had statistically similar properties each day and had stable information for over a week. They proposed that dynamic neuronal activity patterns could balance plasticity for learning and stability for memory. Their notion is very important because it could suggest a mechanism by which cell assemblies simultaneously realize the dynamics and stability of neural representation. Grewe et al. (2017) also recorded two-photon images and tracked the dynamics of ensembles of more than a hundred of amygdala neurons during fear learning and extinction over 6 days. They found that the reshaping of the neural ensemble representation of the conditioned stimulus (CS) became more similar to the unconditioned stimulus (US) representation after conditioning, and the CS representation became more distinctive without reverting to its original form during extinction training. They concluded that these findings support a supervised learning model in which the activation of US representation guides the transformation of CS representation. This study also indicates that the technique is effective to observe the transformation occurred over time in ensemble activity of neurons that are part of the same cell assembly.

Another recent work (Wilson et al., 2016) using two-photon imaging of individual neurons in primary visual cortex has revealed that summation of synaptic inputs can predict the given neuron’s orientation selectivity; however, cannot accurately or reliably predict differences across neurons. This work suggests that there is a significant contribution of non-linearity within the input-output relationship of cell assemblies encoding fundamental properties of the visual world. The result indicates that there are additional characteristics of cell assemblies representing concepts and is related to precise synchrony of firing of neurons, beyond those captured in Figure 3, an extremely simple illustration of functional connectivity of neurons comprising a cell assembly.

Progress for this technique is continuing (e.g., Song et al., 2017), and time resolution will definitely show improvements. Even the low sampling rate at present, particularly if the imaging is of calcium, can indeed allow for burst detection with very high calcium influx to cells (e.g., Vogelstein et al., 2009). This means that although single-neuron spikes cannot be accurately monitored in real time, the synchronous burst firing of many cells constituting a cell assembly can be monitored by this method. The combinatorial application of multineuronal recording and live-cell imaging to respectively monitor the spikes of smaller groups of neurons and the burst firing of larger populations of neurons will be an ideal recoding technique for cell assembly research using behaving animals.

## What and How Cell Assemblies Encode

It is very likely that the cell assembly codes individually stored information, i.e., memory engram as the optogenetics studies have shown, which can create unique functions of memory such as recall and association. However, what needs to be further clarified at present and in the future is what and how the cell assembly actually codes in memory function. The idea that individual cell assemblies code individual and unimodal information is the easiest to understand. For example, the research of Miyashita’s Lab introduced in “Experimental Detection 1” (Takeuchi et al., 2011; Hirabayashi et al., 2013) clearly demonstrates the existence of a cell assembly that codes each of the held visual (figure) information and the recalled figure information.

**Figure 7:**
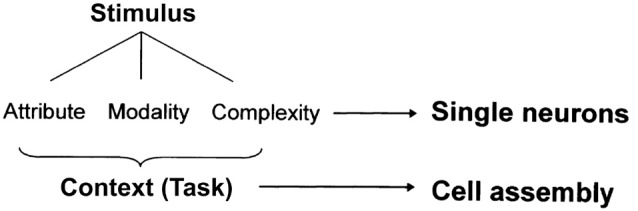
A hypothesis of dual coding by single neurons and cell assemblies. Modified from Sakurai (1999) with permission.

However, it is also certain that the cell assembly codes various information from individual sensory inputs to a “context” that encompasses multiple sensory inputs in an environment. In our previous research (Sakurai, 1996, 2002), as discussed above, the cell assembly coded contextual information, i.e., tasks (Figure 1). In the reactivation experiment of cell assemblies (Liu et al., 2012; Ohkawa et al., 2015) introduced in “Experimental Detection 2” the context information was surely encoded precisely according to the name of conditioning. Sensory input and context are different pieces of information that can be encoded and stored jointly or separately in the neural population. We should further investigate the possibility of shared coding among different cell assemblies and double coding between cell assemblies and single neurons to reveal the joint and separate coding of sensory inputs and context (Sakurai, 1999; Figure 7). This investigation can be performed by observing and analyzing changes in the synchronous firings of neurons that constitute various cell assemblies and in the firing frequency of individual neurons within each cell assembly.

**Figure 8:**
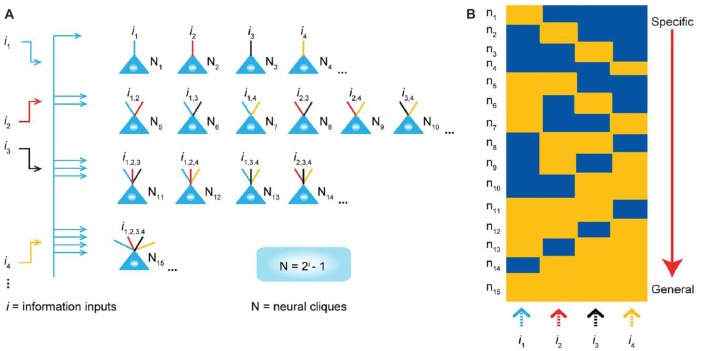
“Power-of-two based permutation logic” for governing the specific-to-general wiring and computational logic of cell assemblies. **(A)** The equation defines the size of a cell assembly; the numbers of neural cliques within a cell assembly. The specific-to-general neural cliques shown in this subpanel illustrate the logic for wiring non-recurrent networks (e.g., the hippocampal CA1). **(B)** Schematic “bar-code” illustrates the specific-to-general cell-assembly activation patterns, which can be measured by electrodes or imaging techniques, from the 15 distinct neural cliques (*N*_1–15_), processing four distinct inputs (*i*_1_, *i*_2_, *i*_3_, *i*_4_). The orange color represents the stimulus-triggered activation above the baseline state (in blue). The arrow on the right side illustrates the number of distinct neural cliques exhibiting specific, sub-combinatorial, as well as generalized, responsiveness. Specific neural cliques encode specific features, whereas various permutation rule-based neural cliques encode various convergent patterns, representing relational memories and generalized concepts. From Xie et al. (2016a) with permission.

Although this review article discusses experimental methods for the detection of cell-assembly activity, it should be noted that theoretical and computational consideration and models based on experimental data are needed to make advance of research of clarifying what a cell assembly or cell assemblies encode. Huyck and Passmore (2013) comprehensively introduce the previous experimental data supporting the existence of cell assemblies and discusses the traditional and recent theoretical models of cell assemblies especially in relation to memory functions. Recently, Tsien and his colleagues have published the series of intriguing theoretical consideration based on their experimental data and suggest the attractive models of cell-assembly coding (Tsien, 2015; Li et al., 2016; Xie et al., 2016a). Their “theory of connectivity” proposes a mathematical rule in organizing the microarchitecture of cell assemblies into the specific-to-general computational primitives that enable memories and adaptive behaviors to emerge in the brain (Tsien, 2015; Li et al., 2016). The theory specifies that within each computational building block, termed “functional connectivity motif” (FCM), the total number of principal projection-cell cliques with distinct inputs should follow the “power-of-two-based permutation logic” (Xie et al., 2016a; Figure 8). Each FCM consists of principal projection neuron cliques receiving specific inputs, as well as other principal projection neuron cliques receiving progressively more convergent inputs that systematically cover every possible pattern using the power-of-two-based permutation logic (Figure 8A).

### Multiple Methods of Approach

Currently, no single measurement method can directly and precisely detect the actual activity of the cell assembly. Multineuronal recording, optogenetics and live-cell imaging, as discussed above, have both advantages and disadvantages. Multineuronal recording has time resolution that can detect neuron spike as it is, and it can record from any part of the brain. However, it is impossible to record all neurons constituting the cell assembly and the method is limited only for the partial sampling of the cell assembly. On the other hand, optogenetics can verify the causal relationship between the activity of the cell assembly and the behavior change but cannot detect the dynamic changes of the cell assembly. Finally, in live-cell imaging, the entire cell assembly can be visualized and changes can be measured in real time. However, the time resolution is insufficient and accurate synchronization between neurons, which is an important property of a cell assembly, cannot be detected.

**Figure 9:**
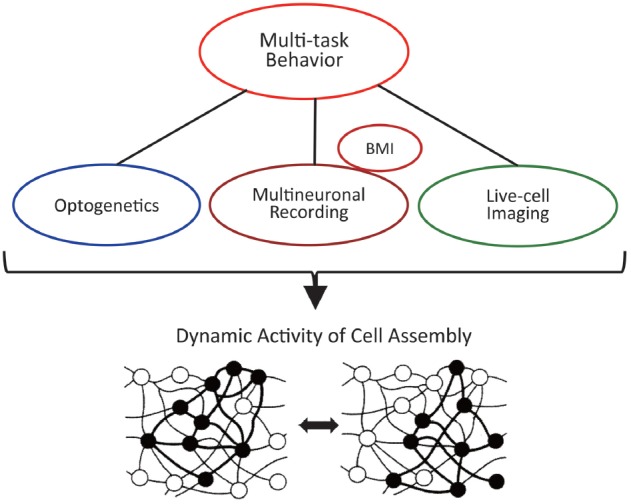
Multiple methods of research to clarify the actual dynamic activity of the cell assembly.

Therefore, it is probably the best strategy to combine and utilize the advantages of these different methods (Figure 9). First of all, the technology that is prerequisite for all measurements is the training of animals to perform multiple tasks, i.e., multiple-task behavior. The technique of operant conditioning (Reynolds, 1975) is particularly indispensable for the training of complicated tasks. By connecting multineuronal recording, optogenetics, and live-cell imaging to that technology, the cell assembly should reveal its actual activity and real nature. Specifically, it is desirable to conduct multineuronal recording and live-cell imaging for the same animal performing multiple behavioral tasks. The former detects real spikes of many neurons and their dynamic changes and the latter visualizes spatial distribution of firing neurons and its transition according to changes of the behavioral tasks (see Harris et al., 2016 for more comparison of live-cell imaging and multineuronal recording). Although, at present, such a plan of applying the two methods simultaneously to the same behaving animal may be only imaginary, it deserves consideration of devising a possible experiment. Using some symmetrical structures in the hemispheres might be an idea to apply the two methods simultaneously, e.g., recording multineuronal activity from the right hippocampus and observing live-cell imaging in the contralateral hippocampus. As anatomical symmetry does not necessarily mean any functional symmetry and the functional “asymmetry” of the hippocampus has been reported in several studies (e.g., Sakaguchi and Sakurai, 2017), functional symmetry and exact synchronization of activity of the right and left hippocampi should be confirmed before the experiments.

Besides the combination of multineuronal recording and live-cell imaging, it is also desirable to conduct optogenetics with them to verify the causal relationship between the activity of neuronal populations and behavioral changes in the multiple tasks. Although the simultaneous application of optogenetics and live-cell imaging is being developed but not for behaving animals yet (e.g., Bovetti et al., 2017), the combination of optogenetics and multineuronal recording is being applied for behaving animals. An excellent example of such studies is Stark et al. (2013), which employed optogenetics and multineuronal recording with multi-contact silicon probes and used optogenetic activation to trigger spiking in pyramidal cells or parvalbumin (PV) interneurons in the hippocampus and neocortex of freely behaving rodents. They showed a causal relationship between activity of PV interneurons and pyramidal cell spiking resonance in the intact cortical networks, elucidating a mechanism of hippocampal theta rhythm related to the cell-assembly coding.

In addition, brain–machine interfaces (BMIs) can be very effective methods for verifying the authenticity and accuracy of the information coded by the cell assembly detected by multineuronal recording on the basis of the evaluation of the movements of the machines being operated by the activity of multiple neurons (Lebedev and Nicolelis, 2011; Lebedev, 2014). As the final goal of a BMI is to detect neuronal activity representing information in the brain, BMI research inevitably faces the problem of neural coding in the working brain (Sakurai, 2014). The early studies of BMIs (Chapin et al., 1999; Wessberg et al., 2000) have already produced very important findings of the neuronal coding, indicating that kinematic and kinetic parameters are coded not by the activities of specific motor-related neurons but by the activity of many distributed neurons in the motor cortex. Subsequent BMI studies supported this notion of neuronal coding (e.g., Carmena et al., 2003) and indicate, as Nicolelis (2003) and Nicolelis and Lebedev (2009) have suggested, that the BMI studies provide significant insights on the actual state of dynamic coding by cell assemblies. At the same time, the theory of cell assembly can be further verified by BMI studies. We should recognize that the origin of research motivation of Dr. Nicolelis, one of the pioneers of BMI research, is the cell assembly (Nicolelis et al., 1997; Nicolelis and Lebedev, 2009).

Another interesting topic of BMIs related to research of cell assemblies and memory is the “hippocampal memory prosthesis,” a brain-machine interface device developed for restoring or enhancing memory functions (Song et al., 2006). It is designed to circumvent damaged hippocampal tissue by reestablishing the ensemble coding of neuronal spikes performed by a normal population of hippocampal neurons (Song and Berger, 2015) and the objective is to restore memory function using nonlinear dynamical models (Song and Berger, 2015). Such an objective requires artificial reconstruction of functional connections of neurons in a way that can be recognized by the remaining normal circuitry, leading to promotion of appropriate interactions of neurons and artificial reconstruction of the actual dynamic coding by cell assemblies. For example, Berger et al. (2011) and Hampson et al. (2012) showed that ensembles of CA3 and CA1 hippocampal neurons, recorded from rats performing a memory task, exhibited successful encoding of trial specific information of events in the form of different spatiotemporal firing patterns. Those patterns, extracted by a specially designed nonlinear mathematical model, were used to predict successful performance online via a closed loop paradigm. The significance of their model as a neural prosthesis has been demonstrated by substituting trains of electrical stimulation pulses to mimic those same ensemble firing patterns. This type of integrated experimental-modeling studies gives us much information about the neural coding of memories and contributes to the progress of research of cell assemblies.

## Conclusion

The number of citations of Hebb’s original book (Hebb, 1949) is still increasing, and reviews of cell assemblies have been constantly published (e.g., Harris, 2005; Buzsáki, 2010; Sakurai et al., 2013; Eichenbaum, 2017). Furthermore, studies of neuronal activities and oscillation of electroencephalograms, in which articles have been explosively increasing over the past decade, are also often considered in relation to cell assemblies (e.g., Buzsáki and Draguhn, 2004). Cell assembly is likely to be a basic unit of higher brain function. For studies trying to elucidate the mechanisms of memory including the association and transformation of information, it is no exaggeration to say that the cell assembly is a central dogma.

## Author Contributions

YS wrote the article based on discussion with YO, YT, EI, JH and HM. All authors reviewed the draft of article.

## Conflict of Interest Statement

The authors declare that the research was conducted in the absence of any commercial or financial relationships that could be construed as a potential conflict of interest.

## References

[B1] Abeles M. (1988). “Neural codes for higher brain functions”, in Information Processing by the Brain. Views and Hypotheses From a Physiological-Cognitive Prespective, ed. Markowitsch H. J. (Toronto, ON: Hans Huber), 225–238.

[B2] Aertsen A. M. Gerstein G. L. Habib M. K. Palm G. (1989). Dynamics of neuronal firing correlation: modulation of “effective connectivity”. J. Neurophysiol. 61, 900–917. 10.1152/jn.1989.61.5.900 2723733

[B3] Berger T. W. Hampson R. E. Song D. Goonawardena A. Marmarelis V. Z. Deadwyler S. A. (2011). A cortical neural prosthesis for restoring and enhancing memory. J. Neural Eng. 8:046017. 10.1088/1741-2560/8/4/046017 21677369 PMC3141091

[B4] Bovetti S. Moretti C. Zucca S. Dal Maschio M. Bonifazi P. Fellin T. (2017). Simultaneous high-speed imaging and optogenetic inhibition in the intact mouse brain. Sci. Rep. 7:40041. 10.1038/srep40041 28053310 PMC5215385

[B5] Buzsáki G. (2010). Neural syntax: cell assemblies, synapsembles, and readers. Neuron 68, 362–385. 10.1016/j.neuron.2010.09.023 21040841 PMC3005627

[B6] Buzsáki G. Draguhn A. (2004). Neuronal oscillations in cortical networks. Science 304, 1926–1929. 10.1126/science.1099745 15218136

[B7] Carmena J. M. Lebedev M. A. Crist R. E. O’Doherty J. E. Santucci D. M. Dimitrov D. F.. (2003). Learning to control a brain-machine interface for reaching and grasping by primates. PLoS Biol. 1:e42. 10.1371/journal.pbio.0000042 14624244 PMC261882

[B8] Chapin J. K. Moxon K. A. Markowitz R. S. Nicolelis M. A. (1999). Real-time control of a robot arm using simultaneously recorded neurons in the motor cortex. Nat. Neurosci. 2, 664–670. 10.1038/10223 10404201

[B9] Denk W. Svoboda K. (1997). Photon upmanship: why multiphoton imaging is more than a gimmick. Neuron 18, 351–357. 10.1016/s0896-6273(00)81237-4 9115730

[B10] Diba K. Amarasingham A. Mizuseki K. Buzsáki G. (2014). Millisecond timescale synchrony among hippocampal neurons. J. Neurosci. 34, 14984–14994. 10.1523/JNEUROSCI.1091-14.2014 25378164 PMC4220030

[B11] Dombeck D. A. Harvey C. D. Tian L. Looger L. L. Tank D. W. (2010). Functional imaging of hippocampal place cells at cellular resolution during virtual navigation. Nat. Neurosci. 13, 1433–1440. 10.1038/nn.2648 20890294 PMC2967725

[B12] Dragoi G. Buzsáki G. (2006). Temporal encoding of place sequences by hippocampal cell assemblies. Neuron 50, 145–157. 10.1016/j.neuron.2006.02.023 16600862

[B13] Driscoll L. N. Pettit N. L. Minderer M. Chettih S. N. Harvey C. D. (2017). Dynamic reorganization of neuronal activity patterns in parietal cortex. Cell 170, 986.e16–999.e16. 10.1016/j.cell.2017.07.021 28823559 PMC5718200

[B14] Eddy S. R. (2004). What is a hidden Markov model? Nat. Biotechnol. 22, 1315–1316. 10.1038/nbt1004-1315 15470472

[B15] Eichenbaum H. (1993). Thinking about brain cell assemblies. Science 261, 993–994. 10.1126/science.8351525 8351525

[B16] Eichenbaum H. (2017). Barlow versus Hebb: when is it time to abandon the notion of featuredetectors and adopt the cell assembly as the unit of cognition? Neurosci. Lett. [Epub ahead of print]. 10.1016/j.neulet.2017.04.006 28389238 PMC5628090

[B17] Fingelkurts A. A. Fingelkurts A. A. Kähkönen S. (2005). Functional connectivity in the brain—is it an elusive concept? Neurosci. Biobehav. Rev. 28, 827–836. 10.1016/j.neubiorev.2004.10.009 15642624

[B18] Friston K. J. Frith C. D. Liddle P. F. Frackowiak R. S. J. (1993). Functional connectivity: the principal component analysis of large (PET) data sets. J. Cereb. Blood Flow Metab. 13, 5–14. 10.1038/jcbfm.1993.4 8417010

[B19] Grewe B. F. Gründemann J. Kitch L. J. Lecoq J. A. Parker J. G. Marshall J. D.. (2017). Neural ensemble dynamics underlying a long-term associative memory. Nature 543, 670–675. 10.1038/nature21682 28329757 PMC5378308

[B20] Grienberger C. Konnerth A. (2012). Imaging calcium in neurons. Neuron 73, 862–885. 10.1016/j.neuron.2012.02.011 22405199

[B21] Gruen S. Rotter S. (2010). Analysis of Parallel Spike Trains. Paris: Springer.

[B22] Hampson R. E. Simeral J. D. Deadwyler S. A. (1999). Distribution of spatial and nonspatial information in dorsal hippocampus. Nature 402, 610–614. 10.1038/45154 10604466

[B23] Hampson R. E. Song D. Chan R. H. M. Sweatt A. J. Riley M. R. Gerhardt G. A.. (2012). A nonlinear model for cortical prosthetics: memory facilitation by hippocampal ensemble stimulation. IEEE Trans. Neural Syst. Rehabil. Eng. 20, 184–197. 10.1109/TNSRE.2012.2189163 22438334 PMC3397311

[B24] Harris K. D. (2005). Neural signatures of cell assembly organization. Nat. Rev. Neurosci. 6, 399–407. 10.1038/nrn1669 15861182

[B25] Harris K. D. Quiroga R. Q. Freeman J. Smith S. (2016). Improving data quality in neuronal population recordings. Nat. Neurosci. 19, 1165–1174. 10.1038/nn.4365 27571195 PMC5244825

[B26] Hebb D. O. (1949). The Organization of Behavior—A Neuropsychological Theory. New York, NY: Wiley.

[B27] Hirabayashi T. Takeuchi D. Tamura K. Miyashita Y. (2013). Functional microcircuit recruited during retrieval of object association memory in monkey perirhinal cortex. Neuron 77, 192–203. 10.1016/j.neuron.2012.10.031 23312526

[B28] Holscher C. Munk M. (2009). Information Processing by Neuronal Populations. Cambridge, MA: Cambridge University Press.

[B29] Horwitz B. (2003). The elusive concept of brain connectivity. Neuroimage 19, 466–470. 10.1016/s1053-8119(03)00112-5 12814595

[B30] Huyck C. R. Passmore P. J. (2013). A review of cell assemblies. Biol. Cybern. 107, 263–288. 10.1007/s00422-013-0555-5 23559034

[B31] Lebedev M. A. (2014). Brain-machine interfaces: an overview. Trans. Neurosci. 5, 99–110. 10.2478/s13380-014-0212-z

[B32] Lebedev M. A. Nicolelis M. A. L. (2011). Toward a whole body neuroprosthetic. Prog. Brain Res. 194, 47–60. 10.1016/b978-0-444-53815-4.00018-2 21867793

[B33] LeDoux J. E. (2000). Emotion circuits in the brain. Annu. Rev. Neurosci. 23, 155–184. 10.1146/annurev.neuro.23.1.155 10845062

[B34] Li M. Liu J. Tsien J. Z. (2016). Theory of connectivity: nature and nurture of cell assemblies and cognitive computation. Front. Neural Circuits 10:34. 10.3389/fncir.2016.00034 27199674 PMC4850152

[B35] Liu X. Ramirez S. Pang P. T. Puryear C. B. Govindarajan A. Deisseroth K.. (2012). Optogenetic stimulation of a hippocampal engram activates fear memory recall. Nature 484, 381–385. 10.1038/nature11028 22441246 PMC3331914

[B36] Loftus E. F. (1997). Creating false memories. Sci. Am. 277, 70–75. 10.1038/scientificamerican0997-70 9274041

[B37] Loftus E. F. Loftus G. R. (1980). On the permanence of stored information in the human brain. Amer. Psychol. 35, 409–420. 10.1037/0003-066x.35.5.409 7386971

[B38] Lopes-dos-Santos V. Ribeiro S. Tort A. B. L. (2013). Detecting cell assemblies in large neuronal populations. J. Neurosci. Methods 220, 149–156. 10.1016/j.jneumeth.2013.04.010 23639919

[B39] Lorente de Nò R. (1934). Studies on the structure of the cerebral cortex II. Continuation of the study of the ammonic system. J. Psychol. Neurol. 46, 113–177.

[B40] Matsuo N. (2015). Irreplaceability of neuronal ensembles after memory allocation. Cell Rep. 11, 351–357. 10.1016/j.celrep.2015.03.042 25900079

[B41] Nicolelis M. A. L. (2003). Brain-machine interfaces to restore motor function and probe neural circuits. Nat. Rev. Neurosci. 4, 417–422. 10.1038/nrn1105 12728268

[B42] Nicolelis M. A. L. Fanselow E. E. Ghazanfar A. A. (1997). Hebb’s dream: the resurgence of cell assemblies. Neuron 19, 219–221. 10.1016/s0896-6273(00)80932-0 9292712

[B43] Nicolelis M. A. L. Lebedev M. A. (2009). Principles of neural ensemble physiology underlying the operation of brain-machine interfaces. Nat. Rev. Neurosci. 10, 530–540. 10.1038/nrn2653 19543222

[B44] Ohkawa N. Saitoh Y. Suzuki A. Tsujimura S. Murayama E. Kosugi S.. (2015). Artificial association of pre-stored information to generate a qualitatively new memory. Cell Rep. 11, 261–269. 10.1016/j.celrep.2015.03.017 25843716

[B45] Paré D. Quirk G. J. (2017). When scientific paradigms lead to tunnel vision: lessons from the study of fear. npj Sci. Learn. 2:6 10.1038/s41539-017-0007-4 PMC6171770 30294453

[B46] Ratte S. Hong S. Schutter E. D. Prescott S. A. (2013). Impact of neuronal properties on network coding: roles of spike initiation dynamics and robust synchrony transfer. Neuron 78, 758–772. 10.1016/j.neuron.2013.05.030 23764282 PMC3753823

[B47] Reynolds G. S. (1975). A Primer of Operant Conditioning. Glenview, IL: Scott Foresman.

[B48] Roudi Y. Dunn B. Hertz J. (2015). Multi-neuronal activity and functional connectivity in cell assemblies. Curr. Opin. Neurobiol. 32, 38–44. 10.1016/j.conb.2014.10.011 25463563

[B49] Sakaguchi Y. Sakurai Y. (2017). Left-right functional asymmetry of ventral hippocampus depends on aversiveness of situations. Behav. Brain Res. 325, 25–33. 10.1016/j.bbr.2017.02.028 28235588

[B50] Sakata S. Harris K. D. (2009). Laminar structure of spontaneous and sensory-evoked population activity in auditory cortex. Neuron 64, 404–418. 10.1016/j.neuron.2009.09.020 19914188 PMC2778614

[B51] Sakurai Y. (1996). Hippocampal and neocortical cell assemblies encode memory processes for different types of stimuli in the rat. J. Neurosci. 16, 2809–2819. 10.1523/JNEUROSCI.16-08-02809.1996 8786455 PMC6578752

[B54] Sakurai Y. (1999). How do cell assemblies encode information in the brain? Neurosci. Biobehav. Rev. 23, 785–796. 10.1016/s0149-7634(99)00017-2 10541056

[B52] Sakurai Y. (2002). Coding of auditory temporal and pitch information by hippocampal individual cells and cell assemblies in the rat. Neuroscience 115, 1153–1163. 10.1016/s0306-4522(02)00509-2 12453487

[B53] Sakurai Y. (2014). Brain-machine interfaces can accelerate clarification of the principal mysteries and real plasticity of the brain. Front. Syst. Neurosci. 8:104. 10.3389/fnsys.2014.00104 24904323 PMC4033401

[B57] Sakurai Y. Nakazono T. Ishino S. Terada S. Yamaguchi K. Takahashi S. (2013). Diverse synchrony of firing reflects diverse cell-assembly coding in the prefrontal cortex. J. Physiol. Paris 107, 459–470. 10.1016/j.jphysparis.2013.05.004 23747709

[B55] Sakurai Y. Takahashi S. (2006). Dynamic synchrony of firing in the monkey prefrontal cortex during working memory tasks. J. Neurosci. 26, 10141–10153. 10.1523/JNEUROSCI.2423-06.2006 17021170 PMC6674631

[B56] Sakurai Y. Takahashi S. (2008). Dynamic synchrony of local cell assembly. Rev. Neurosci. 19, 425–440. 10.1515/revneuro.2008.19.6.425 19317181

[B58] Sato M. Kawano M. Yanagawa Y. Hayashi Y. (2016). *in vivo* two-photon imaging of striatal neuronal circuits in mice. Neurobiol. Learn. Mem. 135, 146–151. 10.1016/j.nlm.2016.07.006 27400866

[B59] Schank R. C. (1999). Dynamic Memory Revisited. New York, NY: Cambridge University Press.

[B60] Schneidman E. Berry M. J. II. Segev R. Bialek W. (2006). Weak pairwise correlations imply strongly correlated network states in a neural population. Nature 440, 1007–1012. 10.1038/nature04701 16625187 PMC1785327

[B61] Song D. Berger T. W. (2015). “Hippocampal memory prosthesis,” in Encyclopedia of Computational Neuroscience, eds Jaeger D. Jung R. (New York, NY: Springer), 11–30. 10.1007/978-1-4614-7320-6_558-1

[B62] Song A. Charles A. S. Koay S. A. Gauthier J. L. Thiberge S. Y. Pillow J. W.. (2017). Volumetric two-photon imaging of neurons using stereoscopy (vTwINS). Nat. Methods 14, 420–426. 10.1038/nmeth.4226 28319111 PMC5551981

[B63] Song D. Chan R. H. M. Marmarelis V. Z. Hampson R. E. Deadwyler S. A. Berger T. W. (2006). Nonlinear dynamic modeling of spike train transformations for hippocampal-cortical prostheses. IEEE Trans. Biomed. Eng. 54, 1053–1066. 10.1109/TBME.2007.891948 17554824

[B64] Squire L. R. (1987). Memory and Brain. New York, NY: Oxford University Press.

[B65] Stark E. Eichler R. Roux L. Fujisawa S. Rotstein H. G. Buzsáki G. (2013). Inhibition-induced theta resonance in cortical circuits. Neuron 80, 1263–1276. 10.1016/j.neuron.2013.09.033 24314731 PMC3857586

[B66] Sternson S. M. Roth B. L. (2014). Chemogenetic tools to interrogate brain functions. Annu. Rev. Neurosci. 37, 387–407. 10.1146/annurev-neuro-071013-014048 25002280

[B67] Stevenson I. H. Rebesco J. M. Miller L. E. Körding K. P. (2008). Inferring functional connections between neurons. Curr. Opin. Neurobiol. 18, 582–588. 10.1016/j.conb.2008.11.005 19081241 PMC2706692

[B69] Takahashi S. Anzai Y. Sakurai Y. (2003). Automatic sorting for multi- neuronal activity recorded with tetrodes in the presence of overlapping spikes. J. Neurophysiol. 89, 2245–2258. 10.1152/jn.00827.2002 12612049

[B68] Takahashi S. Sakurai Y. (2009). Sub-millisecond firing synchrony of closely neighboring pyramidal neurons in hippocampal CA1 of rats during delayed non-matching to sample task. Front. Neural Circuits 3:9. 10.3389/neuro.04.009.2009 19753324 PMC2742662

[B70] Takeuchi D. Hirabayashi T. Tamura K. Miyashita Y. (2011). Reversal of interlaminar signal between sensory and memory processing in monkey temporal cortex. Science 331, 1443–1447. 10.1126/science.1199967 21415353

[B71] Tatsuno M. (2015). Analysis and Modelling of Coordinated Multi-Neuronal Activity. New York, NY: Springer.

[B72] Terada S. Sakurai Y. Nakahara H. Fujisawa S. (2017). Temporal and rate coding for discrete event sequences in the hippocampus. Neuron 94, 1248.e4–1262.e4. 10.1016/j.neuron.2017.05.024 28602691

[B73] Tonegawa S. Liu X. Ramirez S. Redondo R. (2015a). Memory engram cells have come of age. Neuron 87, 918–931. 10.1016/j.neuron.2015.08.002 26335640

[B74] Tonegawa S. Pignatelli M. Roy D. S. Ryan T. J. (2015b). Memory engram storage and retrieval. Curr. Opin. Neurobiol. 35, 101–109. 10.1016/j.conb.2015.07.009 26280931

[B75] Tsien J. Z. (2015). A postulate on the brain’s basic wiring logic. Trends Neurosci. 38, 669–671. 10.1016/j.tins.2015.09.002 26482260 PMC4920130

[B76] Vetere G. Kenney J. W. Tran L. M. Xia F. Steadman P. E. Parkinson J.. (2017). Chemogenetic interrogation of a brain-wide fear memory network in mice. Neuron 94, 363.e4–374.e4. 10.1016/j.neuron.2017.03.037 28426969

[B77] Vogelstein J. T. Watson B. O. Packer A. M. Yuste R. Jedynak B. Paninski L. (2009). Spike inference from calcium imaging using sequential Monte Carlo methods. Biophys. J. 97, 636–655. 10.1016/j.bpj.2008.08.005 19619479 PMC2711341

[B78] Wallace D. J. Kerr J. N. D. (2010). Chasing the cell assembly. Curr. Opin. Neurobiol. 20, 296–305. 10.1016/j.conb.2010.05.003 20545018

[B79] Wess J. Nakajima K. Jain S. (2013). Novel designer receptors to probe GPCR signaling and physiology. Trends Pharmacol. Sci. 34, 385–392. 10.1016/j.tips.2013.04.006 23769625 PMC3758874

[B80] Wessberg J. Stambaugh C. R. Kralik J. D. Beck P. D. Laubach M. Chapin J. K.. (2000). Real-time prediction of hand trajectory by ensembles of cortical neurons in primates. Nature 408, 361–365. 10.1038/35042582 11099043

[B81] Wilson D. E. Whitney D. E. Scholl B. Fitzpatrick D. (2016). Orientation selectivity and the functional clustering of synaptic inputs in primary visual cortex. Nat. Neurosci. 19, 1003–1009. 10.1038/nn.4323 27294510 PMC5240628

[B82] Wolff S. B. E. Grundemann J. Tovote P. Krabbe S. Jacobson G. A. Müller C.. (2014). Amygdala interneuron subtypes control fear learning through disinhibition. Nature 509, 453–458. 10.1038/nature13258 24814341

[B84] Xie K. Fox G. E. Liu J. Lyu C. Lee J. C. Kuang H.. (2016a). Brain computation is organized via power-of-two-based permutation logic. Front. Syst. Neurosci. 10:96. 10.3389/fnsys.2016.00095 27895562 PMC5108790

[B83] Xie K. Fox G. E. Liu J. Tsien J. Z. (2016b). 512-channel and 13-region simultaneous recordings coupled with optogenetic manipulation in freely behaving mice. Front. Syst. Neurosci. 10:48. 10.3389/fnsys.2016.00048 27378865 PMC4905953

[B85] Yoshii T. Hosokawa H. Matsuo N. (2016). Pharmacogenetic reactivation of the original engram evokes an extinguished fear memory. Neuropharmacology 113, 1–9. 10.1016/j.neuropharm.2016.09.012 27639988

